# Wilson disease patient with rare heterozygous mutations in ATP7B accompanied by distinctive nocturnal enuresis: A case report

**DOI:** 10.1097/MD.0000000000020997

**Published:** 2020-07-10

**Authors:** Shijie Zhang, Liangyong Li, Jiuxiang Wang

**Affiliations:** a Experimental Center of Clinical Research; b Department of Neurology, The First Affiliated Hospital of Anhui University of Chinese Medicine, Hefei, Anhui, China.

**Keywords:** ATP7B, nocturnal enuresis, Wilson disease

## Abstract

**Introduction:**

Wilson disease (WD) is an autosomal-recessive disorder of copper metabolism, which exhibits various symptoms due to the combination of environmental and genetic factors. Here, we report a WD patient who displayed distinctive symptom of nocturnal enuresis.

**Patient concerns:**

The patient was a 31-year old woman, who recently developed nocturnal enuresis, combined with hand tremors, trouble speaking, and panic disorder at night.

**Diagnosis:**

The patient had been diagnosed with WD by Kayser-Fleischer rings, abnormal copper metabolism, neuropsychiatric symptoms, and magnetic resonance imaging when she was 17. The diagnosis was further confirmed by genetic analysis, which revealed a compound heterozygous mutations in *ATP7B* gene (c.2195T>C and c.3044T>C). The patient exhibited nocturnal enuresis, but the ambulatory electroencephalogram, routine urinalysis, residual urine detection, color doppler ultrasound of kidney, ureter, and bladder all displayed no abnormality.

**Interventions:**

The patient was treated with sodium dimercaptosulphonate, supplemented with Glutathione and Encephalin-inosine.

**Outcomes:**

The urinary copper excretion level decreased gradually, and the nocturnal enuresis was alleviated along with the neuropsychiatric symptoms by copper chelation therapy.

**Conclusion:**

In this study, we proved that variants c.2195T>C and c.3044T>C is involved in pathogenesis of WD, and revealed that nocturnal enuresis may be a symptom of WD.

## 1 Introduction

Wilson disease (WD, OMIM 277900) is a rare autosomal-recessive disorder of copper metabolism, characterized by an abnormal accumulation of copper in liver, brain and other organs. The disease is caused by homozygous or compound heterozygous mutations in *ATP7B*, with an estimated prevalence of 1 in 30000 in most populations and a mutation carrier frequency of 1 in 90 in general population.^[1]^*ATP7B* encodes for the copper-transporting ATPase2 and mediates the synthesis of ceruloplasmin and copper excretion, and is located on the chromosome 13q14.3 encompassing approximately 85 kb in the genome. More than 700 different mutations consisting of substitutions, deletions, insertions, and duplications have been reported, most of which are missense mutations.^[2]^ The popular mutation sites exist with considerably difference among different subpopulations, and it has been shown that the missense mutation c.2333G>T (p.R778L) in exon 8 and c.3207C>A (p.H1069Q) in exon 14 are the most common *ATP7B* mutations in Asia and Europe, respectively.^[3]^

Dysfunction of *ATP7B* results in rapid degradation of copper-free ceruloplasmin and failure of biliary copper excretion, leading to copper accumulation in the liver, brain, cornea, kidney, and other tissues.^[4]^ The individual clinical manifestation is multifarious, with hepatic and neurologic form as the main features. The hepatic form manifests as hepatocyte dysfunction ranging from steatosis to acute liver failure, hepatitis, and fibrosis, and the neurologic form presents as neuropsychiatric symptoms such as tremors, trouble speaking, muscle stiffness, anxiety, and personality changes.^[5]^ Additionally, Kayser-Fleischer rings exist in 66% of diagnosed WD patients due to the accumulation of copper in the cornea,^[6]^ and other symptoms such as renal diseases, cardiomyopathy, arthritis, pancreatitis, and endocrine manifestations have also been described in different cases.^[7]^ Diagnosis of WD is mainly based on the symptoms described above, and liver biopsy and *ATP7B* gene sequencing should be helpful for confirming the diagnosis.^[8]^

In this study, we describe a WD patient with the distinctive symptom of nocturnal enuresis in addition to hand tremors, trouble speaking, and panic disorder. An obvious improvement of nocturnal enuresis and neuropsychiatric symptoms was seen after four weeks of copper chelation therapy. Finally, molecular diagnosis confirmed 2 rare mutations within *ATP7B* gene which may be related to the disease.

## 2 Clinical report

A 31-year-old patient had been suffering from trouble speaking, difficulty writing, hand tremors, and physical imbalance since the age of 17. The patient displayed positive Kayser-Fleischer rings, abnormal copper metabolism, and further magnetic resonance imaging showed abnormal signal shadows in bilateral lenticular nucleus, indicating a diagnosis of WD. After a long persistent administration of D-penicillamine and sodium dimercaptopropane at low dose, the medication was interrupted twice due to pregnancy. The last interruption continued for 18 months (from June 2016 to December 2017), and the aforementioned hand tremors and trouble speaking aggravated from January 2018, accompanied by the new symptom of nocturnal enuresis and panic disorder at night from June 2018. Two month later, she came to the Department of Neurology at our hospital for comprehensive treatment.

**Figure 1:**
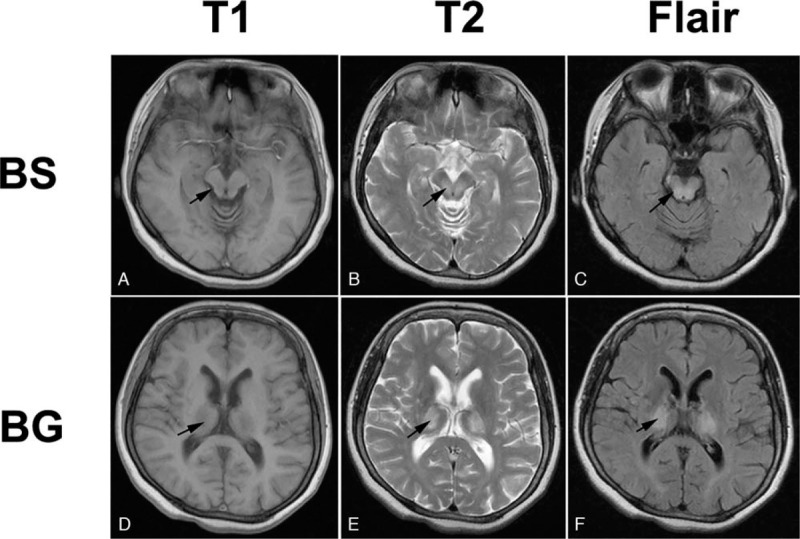
Cranial magnetic resonance imaging image of the patient. Symmetrical patchy signals were observed in brainstem and bilateral basal ganglia in T1-weighted image (A, D), T2-weighted image (B, E), and Flair images (C, F). BS = Brainstem, BG = basal ganglia.

The cranial magnetic resonance imaging showed symmetrical patchy long T1 and long T2 abnormal signals in bilateral basal ganglia and brainstem, and high signals were observed in Flair image (Fig. 1). As shown in Table 1, the biochemical and serological testing revealed an abnormal copper metabolism and a normal liver function, and a normal urinary function was confirmed by routine urinalysis, residual urine detection, and the color doppler ultrasound of kidney, ureter and bladder. Additionally, no abnormalities were detected in ambulatory electroencephalogram. Most importantly, the nocturnal enuresis was relieved along with the improvement of neuropsychiatric symptoms after a four weeks of copper chelation therapy with sodium dimercaptopropane.

**Table 1:**
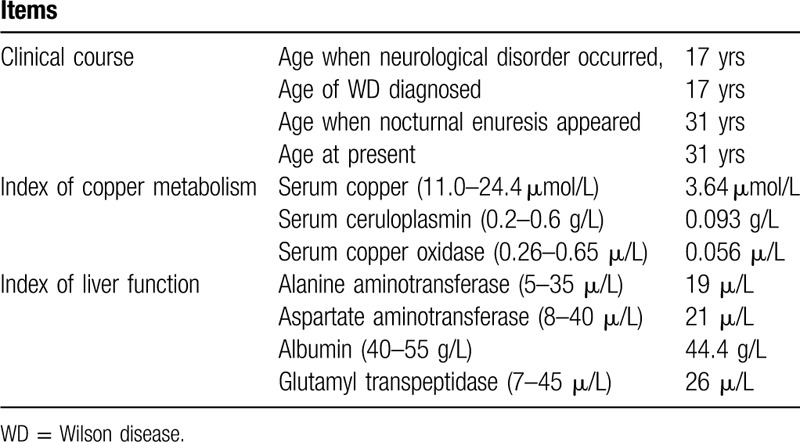
The epidemiological, clinical, and serological features of the WD patient.

## 3 Materials and methods

This study was approved by the Ethics Committee of the First Affiliated Hospital of Anhui University of Chinese Medicine. Informed consent for the publication of this case report was obtained from the patient and related family members.

### 3.1 Genomic deoxyribonucleic acid (DNA) extraction and exons sequencing

The EDTA anticoagulated peripheral blood was collected in the Department of Neurology, the First Affiliated Hospital of Anhui University of Chinese Medicine. Genomic DNA was extracted using method as described previously.^[9]^ Each exon region and the adjacent splice sites of *ATP7B* were amplified by polymerase chain reaction with primers listed in Table 2 and the sequencing was performed using an ABI 3730xl DNA Analyzer.

**Table 2:**
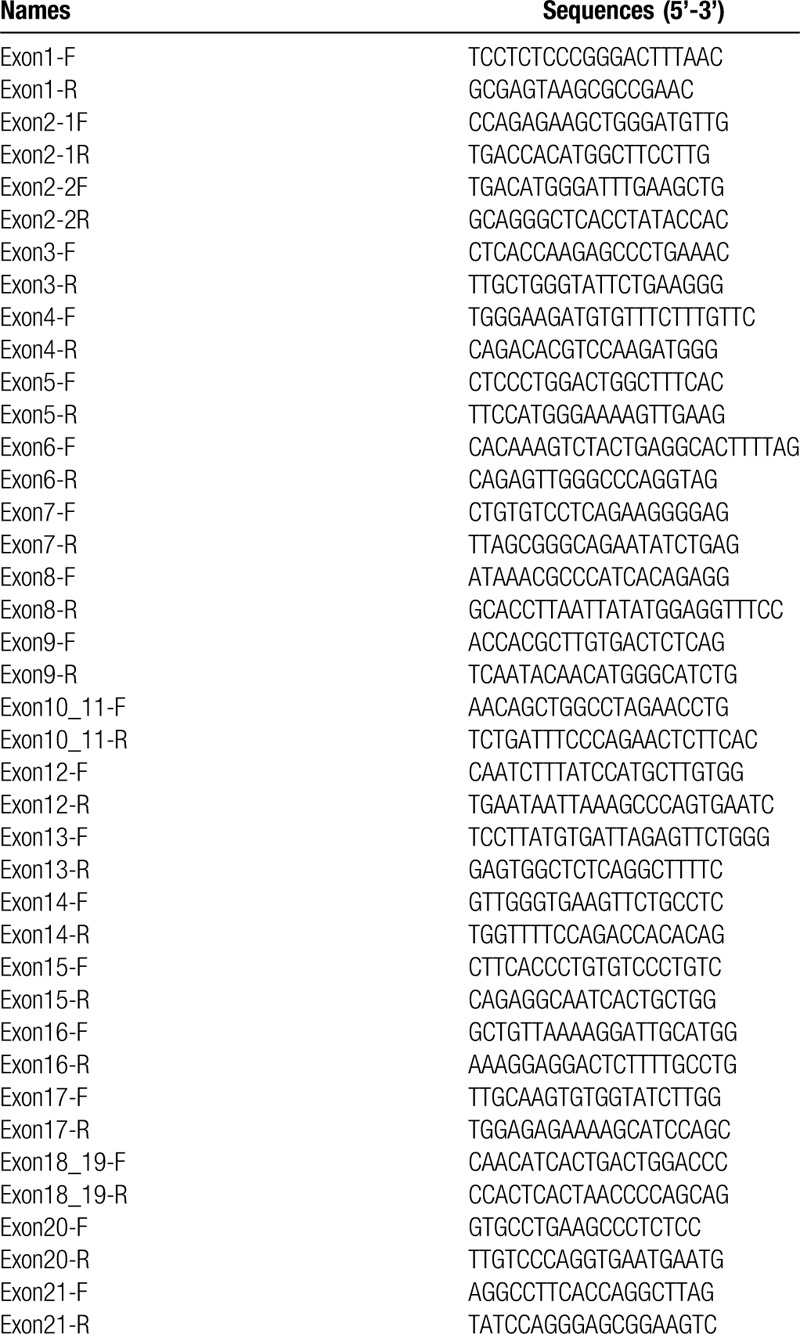
Primers used for amplification of exons and exon-intro boundaries of *ATP7B* gene.

### 3.2 Sequence variants interpretation

The genetic mutations of *ATP7B* were obtained through multiple approaches include Wilson Disease Mutation Databases (http://www.wilsondisease.tk/) and Ensemble Blast/BLAT (http://asia.ensembl.org/index.html). The sequence variants and their minimum allele frequency were interpreted based on the NCBI database SNP (dbSNP) and the 1000 Genomes Project Data. The ATP7B protein sequences of different vertebrates were downloaded from the National Center for Biotechnology Information (NCBI) database and homology comparisons were conducted to explore whether the mutation region is conserved.

## 4 Results

**Figure 2:**
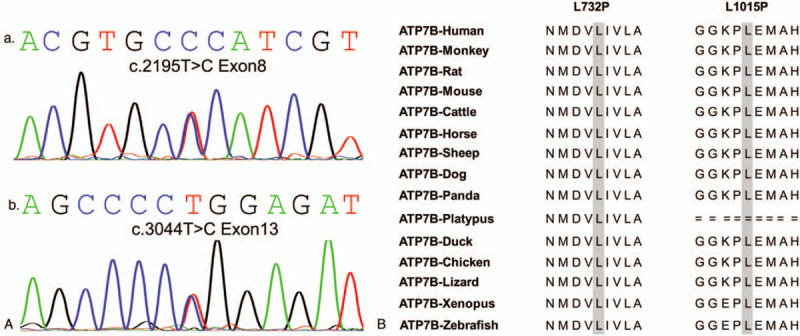
Identification of pathogenic variants within *ATP7B* gene in patient with nocturnal enuresis. (A) The 2 pathogenic variants c.2195T>C (a) and c.3044T>C (b) identified in the patient *ATP7B* gene. (B) Homology comparisons of the mutations in ATP7B protein among 15 species. The 2 variants located within highly conserved regions.

By sequencing of all exons and exon–intron boundaries of the *ATP7B* gene in the patient, 8 alterations were identified (Table 3). The minimum allele frequency of the identified variants in the *ATP7B* gene were determined in the 1000 Genomes Project Data, and only the allele of c.2195T>C and c.3044T>C were less than 0.01. As shown in Figure 2A, the c.2195T>C variant located on exon 8 and c.3044T>C variant located on exon 13 both result in a substitution of leucine (L) to proline (P) (p.L732P and p.L1015P). The 2 variants have been reported in Wilson Disease Mutation Databases and are recorded as rs775151065 and rs1334355798 in dbSNP, respectively.

**Table 3:**
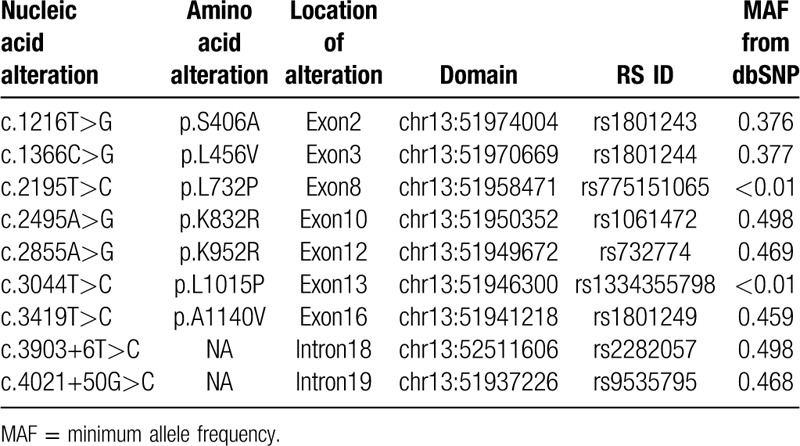
Variants identified in *ATP7B* gene in the WD patient.

According to the ACMG guidelines, variant c.2195T>C can be classed as pathogenic based on evidence of pathogenicity containing PS1, PM2, PP5; variant c.3044T>C can also be classified as pathogenic based on evidence of pathogenicity containing PM2, PP4.^[10]^ However, in previous studies, they existed in different patients and none of them have been reported to be related to nocturnal enuresis.^[10,11]^

## 5 Discussion

Nocturnal enuresis is defined as involuntary voiding during sleep in absence of physical disease, with a prevalence of 0.5% to 2% for adults.^[12]^ However, there has never been any reported connection between nocturnal enuresis and WD patients in previous studies, leaving our identification that nocturnal enuresis is connected to the WD debatable. The pathophysiology of nocturnal enuresis is complicated, revolving around the inter-related mechanisms of overactive bladder, excessive nocturnal urine production, and sleep fragmentation.^[13]^ In this case, the patient initially exhibited common symptoms of WD, and with a long interruption of medication, the distinctive symptoms of nocturnal enuresis appeared. The urinary system and residual urine in the bladder were detected with routine urinalysis, residual urine detection, and the color doppler ultrasound of kidney, ureter and bladder, and displayed no abnormality, excluding the possibility of overactive bladder and excessive nocturnal urine production.

In a previously study, nocturnal enuresis was reported to be associated with fragmented sleep, lower proportions of motionless sleep, and higher nighttime awakening.^[14]^ These are consistent with this patient's panic disorder, which caused the patient to shout and wave her hands at night. The comorbidity between sleep disorders and epilepsy prompted us to conduct an ambulatory electroencephalogram,^[15]^ however the normal results excluded the pathogenic cause of epilepsy. Interestingly, the nocturnal enuresis and neuropsychiatric symptoms were relieved after a comprehensive treatment, which suggests that the nocturnal enuresis was a symptom of WD.

In order to determine the genotype associated with WD's nocturnal enuresis, the exons of *ATP7B* were sequenced and the rare compound heterozygous variants c.2195T>C (p.L732P) and c.3044T>C (p.L1015P) were identified in the patient. The homology comparisons of ATP7B protein in different vertebrates demonstrated that the p.L732P and p.L1015P variants occur within highly conserved regions (Fig. 2B). The p.L732P variant is located on the third transmembrance (TM3) domain of the ATP7B protein, and the p.L1015P variant is located between the TM6 domain and the phosphorylation domain (P-domain) of the ATP7B protein. Based on a bioinformatic method estimating amino acidic change on protein structure, the nonsynonymous SNPs located in TM3 achieved the highest score within the entire protein, followed by the region between TM6 and P-domain.^[16]^ Additionally, the pathogenesis of p.L732P and p.L1015P variants were predicted credible according to the results of SNAP, PolyPhen-2, and SIFT in previously studies.^[10,11]^

WD has multiple phenotypic presentations due to the combined action of genes and environment, and mainly manifests as liver diseases, neurological diseases, or a combination of them. However, it is difficult to associate the phenotypes of the patients with their genotypes, even identical monozygotic twins exhibit different traits.^[17]^ According to Ferenci et al’ study, there was no correlation between *ATP7B* mutations and individual clinical manifestation, whereas a gender and age effect exist.^[18]^ However, research on the relationship between genotypes and specific symptoms have shown several noticeable findings that the popular p.H1069Q variant is related to late onset and neurologic presentation of WD and the compound heterozygous mutations of c.2790_2792del and c.2621C>T is related to premature osteoarthritis.^[19,20]^ Here, we presented one patient carrying the rare compound heterozygous pathogenic variants of c.2195T>C and c.3044T>C, and with progression of the illness, developed the distinctive symptom of nocturnal enuresis.

## 6 Conclusions

In this study, we identified the combination of 2 rare missense variants of p.L732P and p.L1015P in the ATP7B protein that maybe involved in pathogenesis of WD. With the aggravation of the illness, the patient presented with distinctive nocturnal enuresis that has never been reported within WD populations. No urinary system abnormalities were found and the symptoms of the patient were relieved with copper chelation therapy, suggesting that the nocturnal enuresis may be a result of the variants.

## Acknowledgment

We thank the patient and family members for participation in this study.

## Author contributions

**Conceptualization:** Shijie Zhang.

**Data curation:** Shijie Zhang.

**Formal analysis:** Shijie Zhang.

**Funding acquisition:** Shijie Zhang.

**Investigation:** Shijie Zhang.

**Methodology:** Jiuxiang Wang.

**Project administration:** Shijie Zhang.

**Resources:** Liangyong Li.

**Software:** Jiuxiang Wang.

**Validation:** Shijie Zhang.

**Visualization:** Shijie Zhang.

**Writing – original draft:** Shijie Zhang.

**Writing – review & editing:** Shijie Zhang, Liangyong Li.
